# Internal Maxillary Artery-Radial Artery-Middle Cerebral Artery Bypass and STA-MCA Bypass for the Treatment of Complex Middle Cerebral Artery Bifurcation Aneurysm: A Case Report

**DOI:** 10.3389/fsurg.2021.773371

**Published:** 2022-01-24

**Authors:** Chaojue Huang, Shixing Qin, Guan Cao, Wei Huang, Yongjia Yu

**Affiliations:** Department of Neurosurgery, First Affiliated Hospital of Guangxi Medical University, Nanning, China

**Keywords:** pediatric complex aneurysm, middle cerebral artery bifurcation aneurysm, internal maxillary artery, superficial temporal artery, extracranial-to-intracranial bypass, high flow bypass

## Abstract

**Background:**

Children's complex middle cerebral artery (MCA) aneurysm is a relatively rare occurrence. When the huge aneurysm is located in the MCA bifurcation with an inconspicuous neck and involving numerous arteries, intravascular interventional surgery or aneurysm clipping are often difficult treatment options. At this point, high flow bypass revascularization is necessary as a treatment to preserve cerebral blood flow. In recent years, the internal maxillary artery (IMA) has gradually become the mainstream donor artery of thw high flow bypass. We performed internal maxillary artery -radial artery-middle cerebral artery (IMA-RA-MCA) and superficial temporal artery-middle cerebral artery (STA-MCA) bypass as the treatment of a complex MCA bifurcation aneurysm in consideration of the patient's condition and the advantage of the IMA. According to the author, this case is the youngest reported case of IMA-RA-MCA bypass at present.

**Case Description:**

A male child, 7 years and 8 months, was admitted to the hospital due to “recurrent headache for more than 9 months,” DSA indicated that there was a large wide-necked aneurysm at the bifurcation of the right MCA M1 segment, with a size of about 1.16^*^1.58^*^1.32 cm. The inflow path of the aneurysm was in front of M1 bifurcation, and one outflow path originated from the aneurysm body, and another small outflow path attached to the aneurysm body. After completing the preoperative evaluation, an extended pterional approach with zygomatic osteotomy was performed to fully expose the aneurysm and IMA, harvesting the left radial artery at the same time, then a STA-MCA bypass, IMA-RA-MCA bypass, and aneurysm trapping were performed. postoperative re-examination showed that bypass vessels and the distal middle artery vessels were patent and the aneurysm disappeared, the child has no neurological dysfunction.

**Conclusions:**

IMA-RA-MCA bypass is an effective high-flow cerebral blood reconstruct scheme in the treatment of complex middle cerebral artery bifurcation aneurysms. This case can provide a reference for the surgical treatment of complex middle cerebral artery bifurcation aneurysms in children.

## Introduction

Nowadays, intracranial aneurysms are mostly treated by intravascular therapy. Especially in recent years, flow diversion devices (FDDs) have been widely proved to be safe for the treatment of complex cerebrovascular diseases, which is greatly expanding the indications for intracranial aneurysm treatment. However, traditional microsurgery is still difficult to replace in the treatment of MCA M1 bifurcation aneurysms, especially when aneurysms have complex morphology and involve multiple branching arteries.

Complex aneurysms of the middle cerebral artery are rare in children. In this case, the patient was too young and the wide-neck aneurysm was located at the bifurcation of MCA and numerous vessels were involved. Considering long-term prognosis, graft length and other factors, MA-RA-MCA + STA-MCA bypass was performed. According to the author, this case is the youngest reported case of IMA-RA-MCA bypass at present.

## Case Description

### General Information and Preoperative Preparation

**Figure 1:**
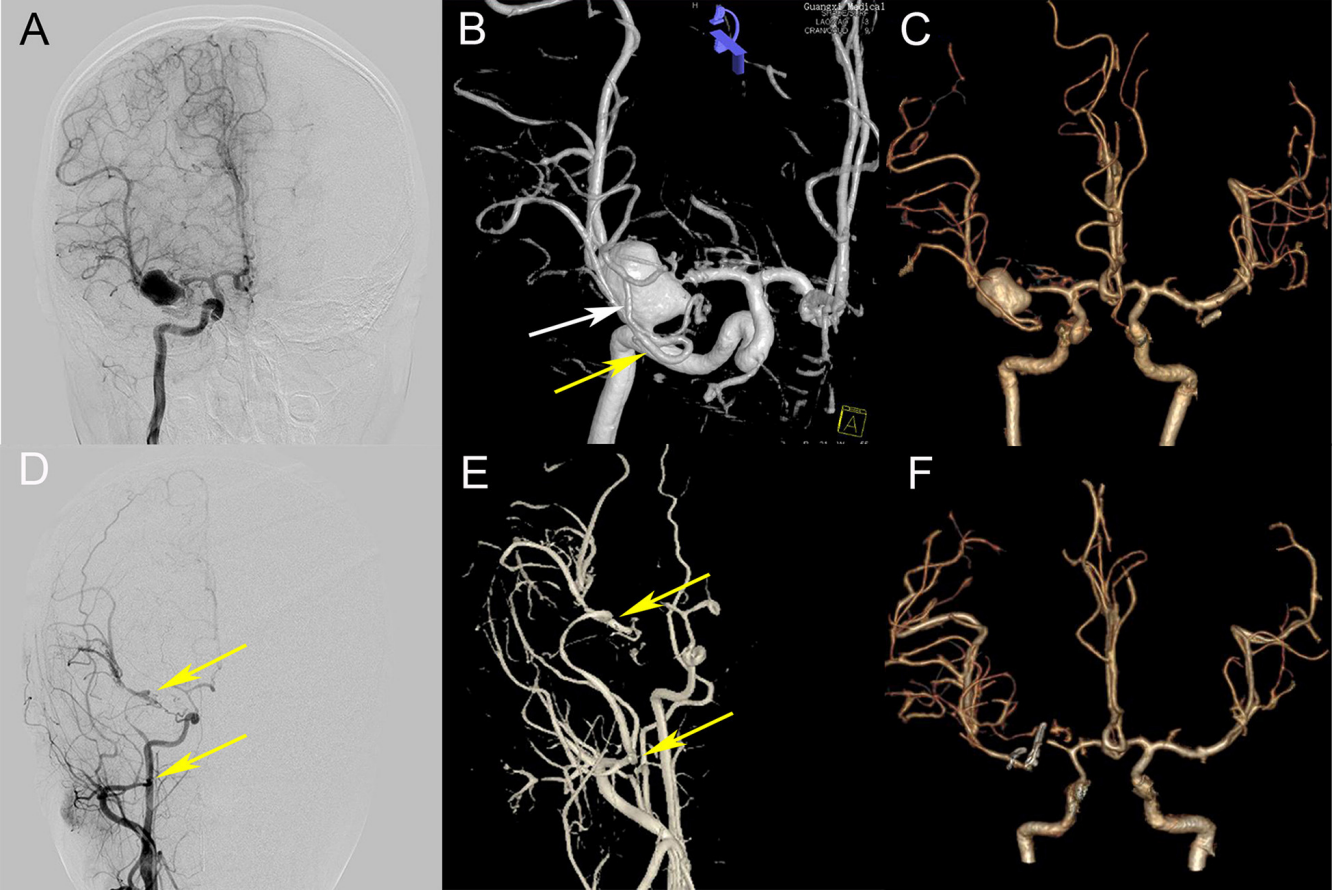
Preoperative and postoperative comparison. **(A,B)** Preoperative DSA (A, frontal view; B, anterior upper view) showing an aneurysm at the bifurcation of M1, a small outflow path (the white arrow) and a larger outflow path (the yellow arrow) at the lateral side of the aneurysm; **(C)** Preoperative CTA 3D reconstruction; **(D,E,F)** Postoperative DSA and CTA showed that the IMX-RA-M2 bypass artery was patent (two yellow arrows indicate the RAG anastomosis), the aneurysm disappeared, and the distal artery of MCA was clearly developed (comparing C and F).

A male child, aged 7 years and 8 months, was admitted to the hospital on April 22, 2021 due to “recurrent headache for more than 9 months” without other complaints of discomfort. About 4 days before admission, the child had a paroxysmal headache, and head CT suggested a high-density shadow in the right sylvian fissure (Possibly meningioma? AVM?) He was transferred to our hospital for further diagnosis and treatment. Cranial nerve and nervous system examination showed no abnormalities. DSA indicated that there was a wide-necked aneurysm at the bifurcation of the M1 segment of the right middle cerebral artery, about 1.16^*^1.58^*^1.32 cm in size. The inflow path of the aneurysm was in the front of M1 bifurcation, and one outflow artery branch was seen to originate from the aneurysm, and another small artery attached to the aneurysm was suspected to be another outflow artery, as shown in Figures 1A,B. Hunt-Hess grade I.

**Figure 2:**
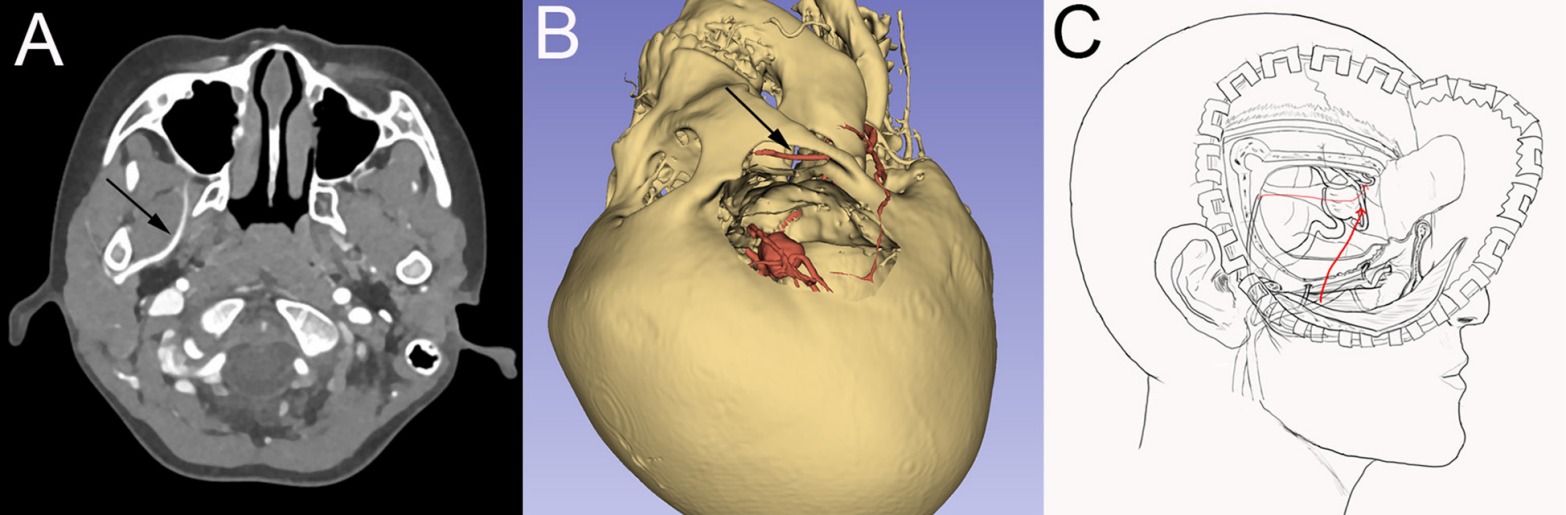
Preoperative examination and surgical design. **(A)** Preoperative CTA showed that the right internal maxillary artery was located deep inside the lateral wing muscle. **(B)** Preoperative 3D display of bone window, aneurysm, and internal maxillary artery, the black arrow is the internal maxillary artery; **(C)** Schematic diagram of bypass surgery.

After discussion by various experts in our ward, the cerebrovascular reconstruction plan was decided, the IMA-RA-MCA bypass and aneurysm trapping were preliminarily proposed after the aneurysm's outflow arteries were adequately explored, STA bypass would be performed when necessary. A preoperative CTA, color ultrasound of the radial artery, and Allen test were completed to evaluate whether the IMA and radial artery were suitable for bypass grafting. The relevant preoperative examination and operation diagram is shown in Figure 2.

### Surgical Procedure

Excluding other surgical contraindication, the extended pterional approach with zygomatic osteotomy was performed, the skull base bone of the inferior temporal fossa was fully bitten to better explore and obtain IMA. The right IMA was explored and exposed with the aid of intraoperative neuronavigation and doppler, the diameter of the IMA was about 2.0 mm, then it was separated and exposed with a length of about 1.5 cm.

**Figure 3:**
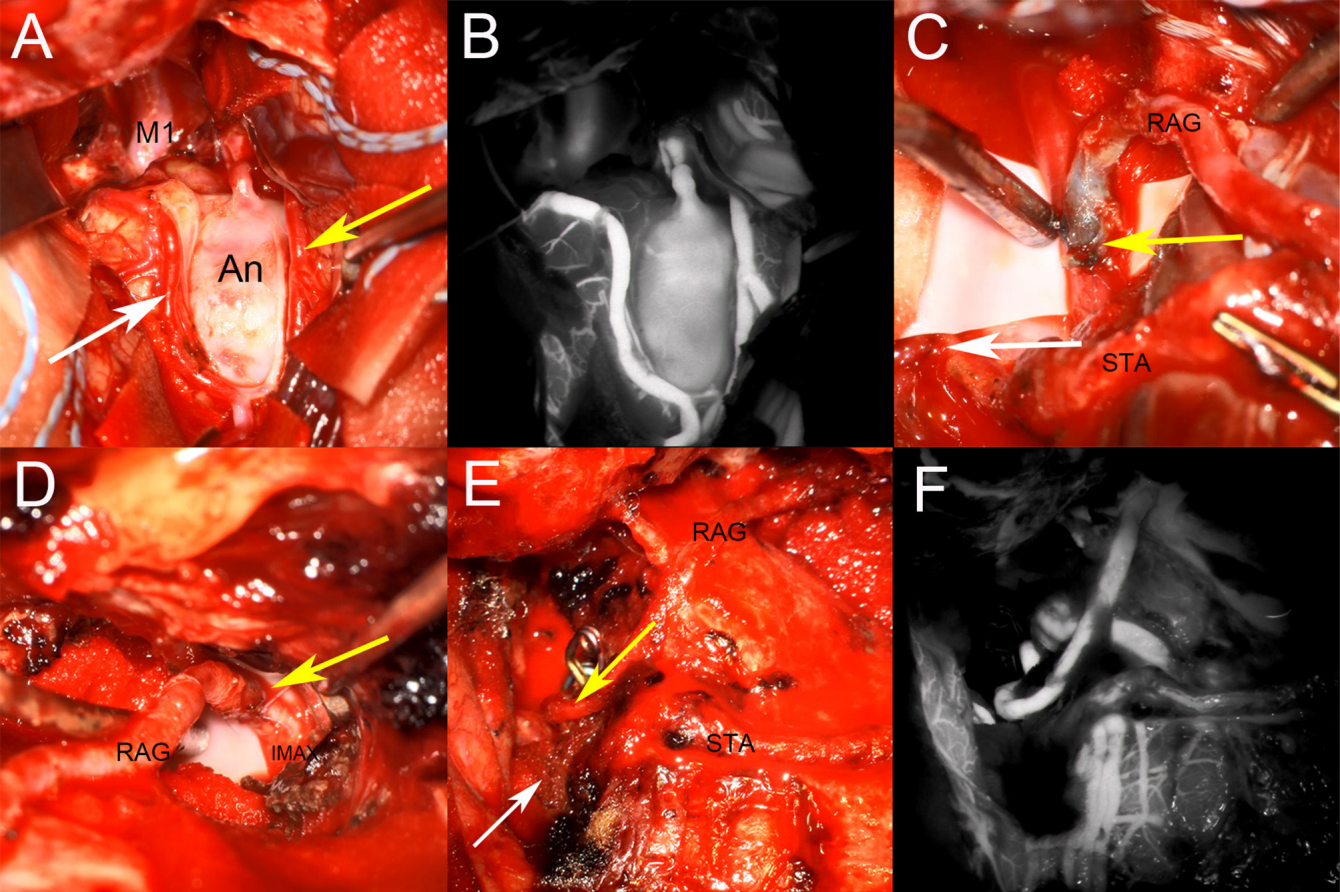
Intraoperative images. **(A)** Aneurysm inflow artery and outflow artery were explored. Two outflow paths were affirmed, location of STA-M2 and RA-M2 anastomosis were indicated by white and yellow arrows; **(B)** Indocyanine green video angiography after aneurysm exploration; **(C)** Arteries conditions after end-to-side anastomosis of intracranial parts, the white arrow indicate STA-M2 anastomosis, and the yellow arrow indicate RA-M2 anastomosis; **(D)** The yellow arrow indicate RA-IMA end-to-side anastomosis; **(E,F)** The aneurysm was isolated after the completion of the bypass, and indocyanine green video angiography showed that the aneurysm disappeared and the reconstructed arteries were patent.

The M1 segment of the right MCA, the aneurysm, anterior cerebral artery, and internal carotid artery were exposed (Figure 1A). The exploration showed that two branches of the MCA emanated from the aneurysm, one of which was larger and emitted two branching arteries, confirming the preoperative conjecture (comparing Figures 3A,B with Figure 1B). During the intracranial operation, the other group of surgeons explored the radial artery of the left forearm. The diameter of the radial artery was about 2.0 mm and the blood flow was patent, 10 cm was cut for use after measuring the distance, rinsing the radial artery lumen with heparin-saline to prevent vasospasm.

The right STA-MCA end-to-side bypass was performed for the smaller outflow path (Figure 3C The white arrow). The recipient artery was blocked for 25 min during the STA-MCA bypass. The end-to-side bypass of the radial artery and the larger outflow path was performed continuously (Figure 3C The yellow arrow). The recipient artery was blocked for 22 min during the RA-M2 bypass. The lateral skull base bone of the inferior temporal fossa was fully bitten when we exposed IMA as mentioned above, RAG exits the right sylvian fissure and follows the surface of the temporal lobe toward the inferior temporal fossa, and the inferior temporal fossa enters to meet IMA through the dura and the skull window of the bottom of the temporal lobe (Figures 3E,F). Then the IMA-RA end-to-side bypass was performed (Figure 3D The yellow arrow), the recipient artery was blocked for 35 min during IMA-RA bypass. The distal part of the IMA was clipped after bypass artery blood flow was evaluated by intraoperative doppler.

Finally, the proximal M1 segment of the aneurysm was separated and exposed again, the outflow path and inflow path of the aneurysm were trapped, respectively. Intraoperative fluorescence angiography showed that the bypass vessels were patent and the aneurysm blood supply disappeared (Figures 3E,F). The aneurysm's blood was continuously blocked for 1 h, and there was no change of motor evoked potential, somatosensory evoked potential or other monitoring indicators in intraoperative electrophysiological monitoring.

During the operation, blood pressure was strictly controlled at the normal level, and 10-0 prolene vascular sutures were used for anastomosis. After bypass, the dura, lateral pterygoid muscle, and temporalis muscle were rested and sutured. The skull flap was properly clipped to avoid excessive compression of bypass arteries and was rested and fixed with the zygoma. Intraoperative images are shown in Figure 3.

### Postoperative Management and Follow-Up

The patient was routinely given intensive care, blood pressure control, and anticoagulant medication as planned after bypass surgery. The postoperative anticoagulant regimen was to take aspirin 50 mg/qd the first day after surgery and take it 6 months postoperatively regularly. Sodium valproate and nimodipine were routinely given to prevent epilepsy and cerebral vasospasm, too. CT before discharge showed no obvious bleeding or infarction. CTA and CTP showed improved blood perfusion in the right MCA supplying area, the distal trunk and branches of the MCA were also well-developed, as shown in Figures 3E,F. MRI indicated a small cerebral infarction in the external capsule area, but the child had no obvious neurological dysfunction.

The child complained of no neurological dysfunction and the daily life was the same as before in follow-up 6 months after the operation. He only had slight discomfort of his left fingers, and often subconsciously gripped the left finger to relieve the symptoms, but there was no paleness of extremities, numbness, or other symptoms, mRS = 1. DSA was re-examined 6 months after the operation, the IMA-RA-M2 bypass were patent and RAG diameter slightly increased than before, and the rest were roughly the same as the DSA 3 months before.

## Discussion

As to complex aneurysms located in the MCA bifurcation, considering the aneurysm wide-neck, involving potential lenticulostriate arteries and multiple branching arteries, conventional aneurysm clipping and endovascular treatment is often difficult to completely resect or isolate the aneurysm, and safeguard cerebral blood flow at the same time. So cerebral vascular reconstruction surgery is very necessary for such a circumstance.

In terms of the selection of reconstruction methods, Due to the complex vascular route of the aneurysm at the MCA bifurcation, it is generally accepted that middle or high flow bypass should be performed in the need of reconstructing the blood supply from MCA. Lawton consider that middle or high flow bypass is the preferred treatment for MCA bifurcation aneurysms (1). Combined bypass is a combination of two or more bypass procedures based on the six single bypass procedures summarized by Lawton et al. requiring more than two intraoperative anastomoses, such as double branch replantation using extracranial blood supply branch, double branch replantation using intracranial blood supply branch, IC-IC bypass + EC-IC bypass, etc. For unruptured MCA bifurcation aneurysms, single EC-IC high-flow bypass, or double STA- MCA bypass (2–4) can also be used as reconstruction schemes.

Combined with the characteristics of preoperative examination in this case, the risk of aneurysm clipping or interventional embolization is high on account of the bifurcation of MCA in children is complicated and the diameter is small. Moreover, considering the long-term prognosis and the risk of aneurysm rupture, aneurysm trapping is the first choice. In addition, as the most commonly used extracranial blood supply artery, the blood flow provided by STA is related to the diameter and the recipient artery of the intracranial artery (2). Successful treatments of STA-MAC bypass that can provide blood flow >100 mL/min have been reported previously (2, 3, 5, 6), but the anterior and parietal branches of STA are relatively thin in this case, and the diameter is about 0.5 mm, so it is difficult to replace blood supply effectively if a double STA-MCA bypass is performed alone (5). Therefore, STA can only be used as a secure blood supply artery, and cannot replace the role of a high-flow blood supply artery. It should be noted that in this case, STA was not clearly displayed in postoperative angiography (Figures 1D,E), which was considered for two reasons: (1) the formation of small thrombosis in STA; (2) the blood supply of IMA-RA-M2 had completely replaced distal blood of MAC and the pressure difference at the anastomosis of STA-MCA was too small to display STA. We think the second point is the main reason.

Prior STA-M2 bypass provides partial blood flow to the brain during high-flow bypass artery occlusion, ensuring security when performing the complex and difficult high flow bypass afterwards. Once the high flow bypass failed, the STA can still guarantee part of effective blood artery blood supply to the brain. Therefore, the final plan was STA-M2 bypass first, followed by IMA-RA-M2 bypass, then to finally trap the aneurysm.

Compared with the intracranial insertion bypass, extracranial double-branch replantation of extracranial blood supply, or external carotid artery (ECA)-graft-MCA single-branch high-flow bypass previously, the core characteristic of this case's scheme is using the IMA as a high-flow donor artery for a seven-year-old child, which reflects the advantages of the IMA-RA-MCA bypass. In recent years, with the development of research and technical practice, the advantages of IMA as a high-flow bypass donor artery in the treatment of complex MCA arteries aneurysms have been well-established (7), and there have been many successful cases and case series reports (8–12), confirming that the IMA can be a safe and efficient high-flow donor artery. Compared with ECA, IMA has many advantages such as a single cranial incision, shorter distance from the intracranial recipient artery which reduces the length of the graft, thus reducing the risk of postoperative bypass vessel torsion, and the deeper location can effectively avoid compression, etc (9). Furthermore, the disadvantages are that the position of IMA is deep and difficult to expose, and the technical requirements of the operator are higher. In our case, due to the young age, the length of the RA graft that can be provided is limited. If performing ECA-RA-MCA bypass, it usually needs to intercept 16–18 cm RA graft (13–15). However, the seven-year-old child's short forearm makes it difficult to intercept such a length, and there is a risk of bypass vessel torsion at the same time.

The application of IMA can fully reflect its advantages and effectively solve the problems such as limited graft length (16). Generally, the second and third segments of IMA, namely the pterygoid and the Pterygopalatine segments, are used as bypass donor arteries (17, 18). To better obtain IMA, there have been numerous reports exploring more appropriate approaches and craniotomy techniques to expose IMA in recent years (19–21). Considering the operating space required for bypass surgery, we agree with the experience of Professor Xiang'en Shi's team at Beijing Sanbo Brain Hospital (9, 10, 22), Zygomatic arch was removed and the skull base bone was bitten off when necessary to fully expose the inferior temporal fossa during craniotomy so that the pterygoid segment of IMA can be fully separated. In this case, no serious masticatory muscle-related complications occurred. Preoperative CTA was performed to predict IMA location, and intraoperative neuronavigation and doppler probe assisted detection of IMA helped to obtain IMAX more efficiently and safely.

In terms of graft selection, RA has good matching, convenient for preoperative evaluation and acquisition, and good postoperative patency, but vasospasm and other complications should be paid attention to. Previous studies have pointed out that the RA is not suitable to be used as the graft artery in children younger than 12 years old (9, 16), but there is also case reported that the RA was used as the graft artery in the treatment of MCA in a 4-year-old child (23). In this case, preoperative examination and intraoperative vascular matching showed that RA was suitable for bypass grafting. The diameter of RA was matched with MCA, and postoperative fluorescence angiography showed that the artery was patent. Only 10 cm of RA was needed to effectively avoid postoperative forearm ischemia, and results of postoperative follow-up were good.

In addition, intraoperative electrophysiological monitoring ensures the control of the risk of occlusion and provides a feasible and effective judgment on the blood flow recovery after bypass. Real-time fluorescein angiography after bypass also confirmed patency of blood flow, and preoperative and postoperative CTP comparisons confirmed no severe cerebral ischemia events. Postoperative MRI showed local subacute cerebral infarction in the right external capsule area, but the child had no neurological dysfunction.

Finally, as a case study with a single sample size, and a short follow-up time, this case can still provide a reference for children's complicated aneurysms cases at that age. Larger case studies with long-term follow-up should be conducted in the future to more fully evaluate the advantages of this type of bypass surgery in the treatment of complex MCA aneurysms in children.

## Conclusions

IMA-RA-MCA bypass can be used as an effective high-flow bypass to reconstruct the blood flow at MCA bifurcation, combined with STA-MCA bypass, it can safely treat complex aneurysms at MCA bifurcation. As a high-flow donor artery, IMA has advantages in bypass surgery for children with complex aneurysms of MCA. This case can provide a reference for the treatment of complex aneurysms of MCA bifurcation in children of this age.

## Data Availability Statement

The original contributions presented in the study are included in the article/supplementary material, further inquiries can be directed to the corresponding author/s.

## Ethics Statement

Written informed consent was obtained from the minor's legal guardian/next of kin for the publication of any potentially identifiable images or data included in this article.

## Author Contributions

CH and WH: conceptualization and study design. SQ and GC: data collection, literature research, and manuscript drafting. CH, WH, and YY: revision and funding. All authors contributed to the article and approved the submitted version.

## Funding

This work was supported by the Guangxi Medical and Health Key Scientific Research Project (No. Z20190610).

## Conflict of Interest

The authors declare that the research was conducted in the absence of any commercial or financial relationships that could be construed as a potential conflict of interest.

## Publisher's Note

All claims expressed in this article are solely those of the authors and do not necessarily represent those of their affiliated organizations, or those of the publisher, the editors and the reviewers. Any product that may be evaluated in this article, or claim that may be made by its manufacturer, is not guaranteed or endorsed by the publisher.

## Abbreviations

CT: computed tomography
CTA: CT-angiography
CTP: CT-perfusion
DSA: digital subtraction angiography
MRI: Magnetic resonance imaging
RA: Radial artery
RAG: Radial artery graft
MCA: Middle cerebral artery
IMA/IMAX: Internal maxillary artery
STA: Superficial temporal artery
mRS: modified Rankin Scale
EC-IC: extracranial-to-intracranial
IC-IC: intracranial-to-intracranial
ECA: external carotid artery.
